# Self-reconfigurable robot vision pipeline for safer adaptation to varying pavements width and surface conditions

**DOI:** 10.1038/s41598-022-17858-w

**Published:** 2022-08-25

**Authors:** Lim Yi, Braulio Félix Gómez, Balakrishnan Ramalingam, Madan Mohan Rayguru, Mohan Rajesh Elara, Abdullah Aamir Hayat

**Affiliations:** 1 Engineering Product Development Pillar, Singapore University of Technology and Design (SUTD), Singapore, 487372 Singapore; 2 Electrical Engineering, Delhi Technological University, New Delhi, 110042 India

**Keywords:** Engineering, Electrical and electronic engineering

## Abstract

This work presents the vision pipeline for our in-house developed autonomous reconfigurable pavement sweeping robot named Panthera. As the goal of Panthera is to be an autonomous self-reconfigurable robot, it has to understand the type of pavement it is moving in so that it can adapt smoothly to changing pavement width and perform cleaning operations more efficiently and safely. deep learning (DL) based vision pipeline is proposed for the Panthera robot to recognize pavement features, including pavement type identification, pavement surface condition prediction, and pavement width estimation. The DeepLabv3+ semantic segmentation algorithm was customized to identify the pavement type classification, an eight-layer CNN was proposed for pavement surface condition prediction. Furthermore, pavement width estimation was computed by fusing the segmented pavement region on the depth map. In the end, the fuzzy inference system was implemented by taking input as the pavement width and its conditions detected and output as the safe operational speed. The vision pipeline was trained using the DL provided with the custom pavement images dataset. The performance was evaluated using offline test and real-time field trial images captured through the reconfigurable robot Panthera stereo vision sensor. In the experimental analysis, the DL-based vision pipeline components scored 88.02% and 93.22% accuracy for pavement segmentation and pavement surface condition assessment, respectively, and took approximately 10 ms computation time to process the single image frame from the vision sensor using the onboard computer.

## Introduction

With urbanization, more infrastructure and pavements will be developed around the world. Due to this increase in pavements, more types of pavement are developed. These pavements generally vary in their properties, like surface condition, texture, and wet or waterlogged material, among others. In addition to the increasing number of pavement types, more pavements are required to be maintained daily to ensure a hygienic environment for social activities to occur. In Singapore, 200 km of sheltered pavements have recently been built, and it is expected to have more in the future^1^. A lot of manual labor and resources go into keeping these pavements clean for usage. With the rise of Industry 4.0 and automation, robots are researched extensively to reduce the amount of manual labor required for repetitive tasks, including the pavement cleaning industry.

**Figure 1:**
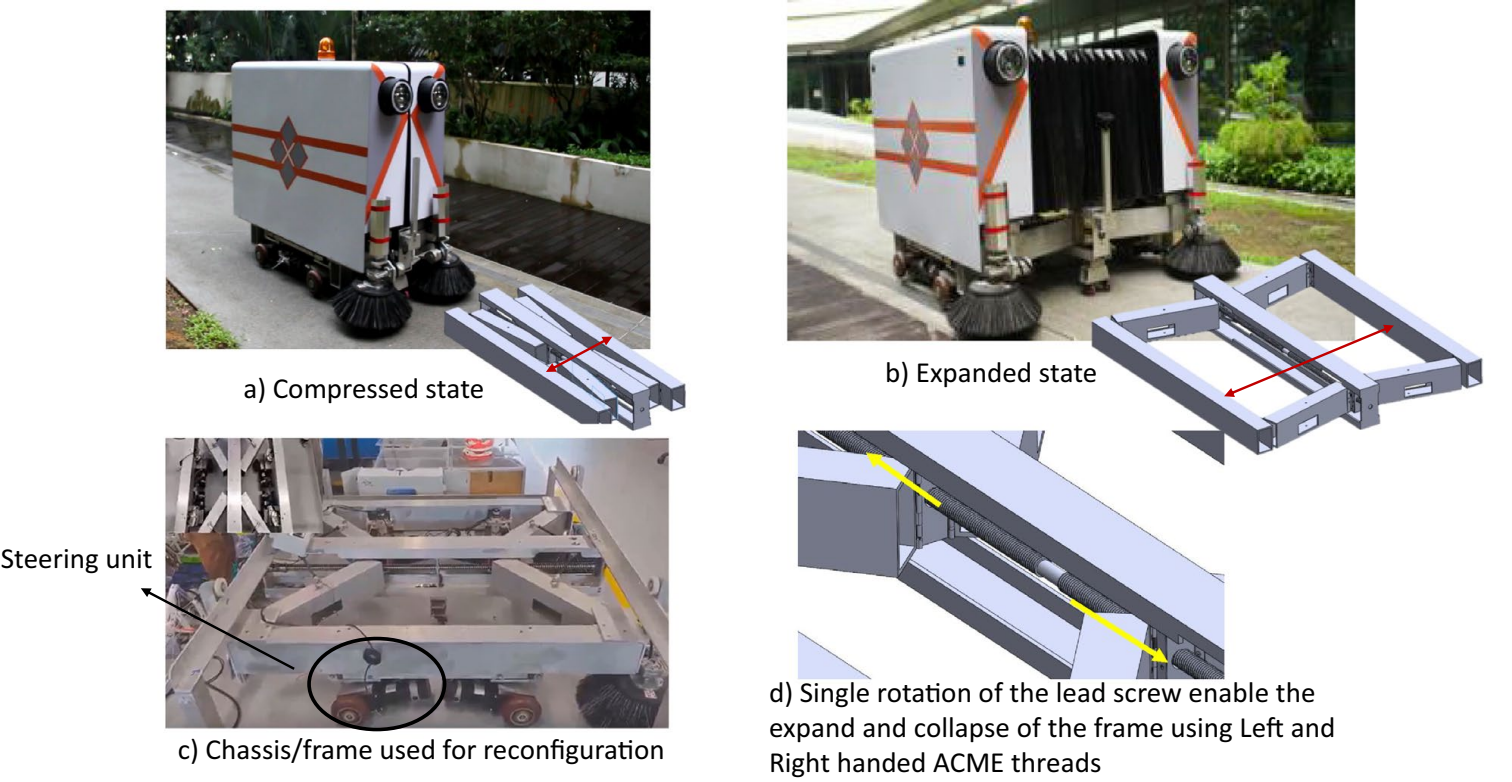
Self-reconfigurable pavement sweeping robot Panthera.

In recent years many autonomous robots^2,3^ and cleaning vehicles^4^ were proposed for sweeping pavement tasks. However, those platforms have a lot of limitations, and is inefficient in covering the width of pavement, and are hard to use in the narrow pavements region. As a result, limited efficiency is achieved during the pavement cleaning tasks. Reconfigurable robots^5^ are becoming a viable alternative for fixed morphology robots. These robots are developed with an inherent capability to autonomously change their kinematics to overcome difficulties in handling a given task and traversing the environment. Purposes of shape reconfiguration varies such as to perform another motion such as climbing^6^, rolling^7^, flying^8^ and floating^9^. By considering the advantage of the reconfigurable mechanism, the pavement cleaning robot Panthera^10,11^, as seen in Fig. 1, was developed with the reconfigurable mechanism. The robot moves through pavements with dynamically changing widths through a reconfigurable mechanism and performs efficient cleaning. To push the development of reconfigurable robot Panthera towards self-configurable capability and safe autonomous locomotion with the instantaneous center of rotation^12^, the kinematic control part of Panthera^13^ needs a high-level vision pipeline that should have the capability to provide the information about changing pavement width, pavement types, and condition of the surface.

To our best of knowledge, Deep learning (DL) based vision pipeline for reconfigurable pavement cleaning robots is a new approach as it is not widely studied yet. This work proposed a DL-enabled vision pipeline for the Panthera robot, which provides essential information such as pavement type, pavement condition, and varying pavement width information for safe and efficient operation. The vision pipeline is built with Deep Convolutional Neural Networks (DCNN) based semantic segmentation algorithm and image classifier module. The DCNN modules are trained with large-sized pavement image datasets, collected from the pavements in Singapore and tested with offline and real-time environments.

### Related work

This section describe the existing study related to our work and its short summery is given in Table 1. Machine learning (ML)^14^ and deep learning (DL)^15^ are emerging techniques which are widely used for many indoor and outdoor robots^16^, autonomous vehicle perceptual system design in recent years. These perceptual system vision pipelines data are widely used for^17^, path planning^18^, controlling , safe navigation^19^ and efficient operation^20^. In ML techniques, support vector machine (SVM), K-nearest neighbors^21^, Bayes classifier, and neural network (NN) are commonly used algorithms and perform surface condition assessment or classify the surface type from images or various sensors data.

**Table 1:** Summary of related work.

Related work	Algorithm	Application	Advantage	Limitation
Liang et al.^29^	D-UNet	Road surface condition prediction	Highest classification performance compared to ML algorithm	Due to pre-trained CNN classification, capacity is limited
Marcus et al.^30^	ResNet50 and InceptionV3	Road friction estimation	Classify six types of surface	Misclassification of wet asphalt and dirt as asphalt
Suryamurthy et al.^32^	Segnet	Terrain segmentation and roughness estimation	Real-time	Bias between flat surface and smooth boundaries

In Khan et al. work^22^, the authors proposed a terrain classification algorithm for mobile robot applications. The features extracted from the mobile robot collected images are used to classify terrain type using the Random Forest (RF) algorithm and scored 99.2% terrain classification accuracy. Omer et al.^23^ investigate the feasibility of classifying winter road surface conditions as bare road, snowy road, and tracks. The authors use the support vector machine (SVM) algorithm, trained using 400 images each class collected through a vision system mounted on regular vehicles and have a classification accuracy of over 80%. Kawai et al.^24^ propose a distinction method for road surface conditions at night. The author uses the differences in image features of dry, wet, and snowy roads under different light sources and combines the three features with color, brightness, and texture of road to classify the road surface condition. K-nearest neighbor algorithm is used for classification and reported 96.1%, 89.4%, and 95.6% classification accuracy, respectively.

In contrast with the ML technique, the DL scheme has a lot of advantages in perceptual system design, which has automatically extracted and learned the features from the bulk image datasets and performs classification and detection accuracy better than ML techniques. In literature, there are many DL based image classification framework such as ResNet^25^, SqueezeNet^26^, MobileNet and VGG16^27^ which were trained and used for autonomous vehicle road pavement classification and condition detection task. In^28^ Ramon et al. assess Neural Network’s (NN) performance, ML, and DL for terrain classification and slip estimation. The authors performed the terrain classification estimation in the mobile robot Fitorobot and reported that deep learning models are optimal for solving terrain and ground robotics problems. In another study, Liang et al.^29^ used the road surface status recognition system using a deep semantic segmentation framework. The author uses the D-UNet encoder–decoder framework for detecting the slippery road statuses caused by water, ice, and snow in the wintertime. Deep Convolutional Neural NetworK-based road friction estimation was proposed by Marcus et al.^30^. Here, the author trained and evaluated two pre-trained models, ResNet50 and InceptionV3^31^, and reported that ResNet50 outperforms InceptionV3 for road friction estimation and classification tasks. Suryamurthy et al.^32^ adopt the deep convolutional encoder–decoder framework in CENTAURO Robot for safe reconfiguration of leg joints and path planning application where the semantic segmentation framework “SegNet” was trained for terrain segmentation and roughness estimation task and obtained 64% classification accuracy.

The previous work^33^, an RGB-D camera is used to estimate the vision feedback algorithm parameters for Panthera locomotion and reconfiguration using VGG16 semantic segmentation. Semantic segmentation used in^33^ was unable to classify different pavement types in Singapore accurately. The parameters derived through the method directly go into Panthera kinematic control^13^ and do not take into account the pavement types, conditions, and magnitude of the vision feedback algorithm parameters for safety. As pre-trained model VGG-16 pavement segmentation cannot perform accurately in Singapore pavements, safety of pavement users and the self-reconfigurable robot is compromised as it might make the robot move into non-pavements. On top of that, there is little or no work that is performed on a speed regulation on a self-reconfigurable robot based on pavement width changes and pavement conditions.

Taking into account the above facts, the following objectives of the present paper as:

- Vision pipeline for semantic segmentation with pavement type classification (concrete, road, and paver block) and condition (bad, moderate, and good) and estimation of pavement width in Singapore.
- A fuzzy-based controller for safer adaptation based on pavement type condition classification and vision feedback control parameters.

The remaining of this paper is structured as follows. In “Panthera overview” section describe the proposed Panthera system and overview. In “Vision pipeline” section discuss the vision pipeline and its modules. In “Fuzzy inference system” section Section will discuss the fuzzy logic control system part. In “Experimental results” section will discuss the experimental results. In “Conclusions” section concludes the paper.

## Panthera overview

Figure 1 shows the overview of the reconfigurable pavement sweeping robot Panthera. It was designed to reconfigure in shape during locomotion to adapt smoothly with respect to changing pavement widths^10,33–35^. The taxonomy of reconfigurable systems and its classification along with the sensor fusion are detailed in the respective works^5,36^. The detail of the mechanical system overview and control system architecture is briefly described next.

### Mechanical overview

The Panthera core frame is made of an aluminum scissors mechanism, and the entire body is supported by four steering units attached to the aluminum frames. These four steering units are independent differential drives and consist of two wheels each. In total, Panthera has eight wheels with eight-wheel motors for locomotion. The mechanism responsible for the reconfiguration is the scissors mechanism that connects the central beam to two side beams. A double-threaded lead screw is connected to a motor which will drive the scissors mechanism to move as seen in Fig. 1d. The movement of the scissors mechanism enables the robot to expand and contract. As the steering units are connected to the aluminum scissors mechanism side beam as seen in 1c, the lead screw motor and steering units have to work in synchronization so that reconfiguration can be performed smoothly. Panthera core frame supports the robot’s electronics, including the batteries, micro-controllers, relays, perspective sensors, and the industrial computer. The hardware and sensor components are sheltered by Panthera’s two external aluminum covers, which are mounted on the two side beams of Panthera. The two external aluminum covers are connected by an artificial leather bellow, water resistance, and protect the internal electronics components from water and other foreign objects. The kinematics of Panthera can be found in our previous work^33^.

### Control system architecture

**Figure 2:**
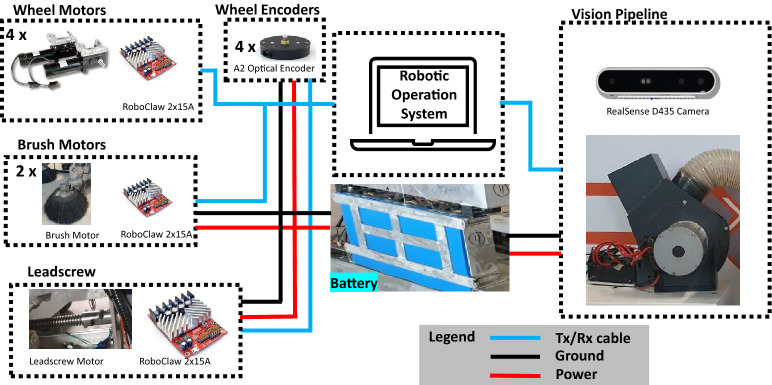
Hardware components and electrical layout.

The Panthera central control system is built on an industrial computer with the operating system Ubuntu 16.04 and uses the middle-ware robot operating system (ROS) Kinetic version. ROS is capable of parallel information transfer between ROS nodes. It is used to publish and subscribe data within Panthera functional components. Figure 2 shows the hardware components of control system architecture. The industrial computer has a GPU, 8CPU core, and 16 GB RAM, performing high-level tasks including running ROS master, vision pipeline task, etc. The 24-V traction battery is used in the Panthera. The 24 V battery unit powers all of Panthera’s motors, including eight-wheel motors, one lead screw motor, two brush motors, and a vacuum motor. It also powers the sensors and other low-level controllers in Panthera. The Panthera has been built with three key sensors for safe locomotion operation: Digital Absolute Encoders, US Digital Incremental Encoders, and the RealSense D435 camera. The RealSense D435 camera is mounted at 95 cm in front of Panthera. The RealSense D435 RGB-D sensor (called perspective nodes) connects with the industrial PC through a USB 3.0 interface. It publishes RGB images and depth images in two topics as a sensor_msgs/Image message and is subscribed by the vision processing node. After subscribing to the information from the perspective nodes, the vision pipeline node will execute the pavement segmentation, pavement classification, surface condition prediction, and pavement width detection task.

Based on the type of pavement classified, surface condition predicted, and pavement width detection, the processing node will then publish three parameters: Pavement classification K factor, beta left, and beta right in a topic. These three parameters will be subscribed by the locomotion and reconfiguration node via a geometry_msgs/Twist message. Furthermore, the US Digital Absolute Encoders provide steering angle feedback for steering angle, while the US Digital Incremental Encoder provides the velocity feedback for the wheel’s speed. Both steering angle feedback and velocity feedback are important for Panthera’s control during locomotion and reconfiguration. The encoder module published geometry_msgs/Twist message and locomotion and reconfiguration node subscribed this topic for locomotion and reconfiguration operation. The inverse kinematics will determine the speed of the leadscrew motor and the wheel motors for reconfiguration during locomotion.

## Vision pipeline

**Figure 3:**
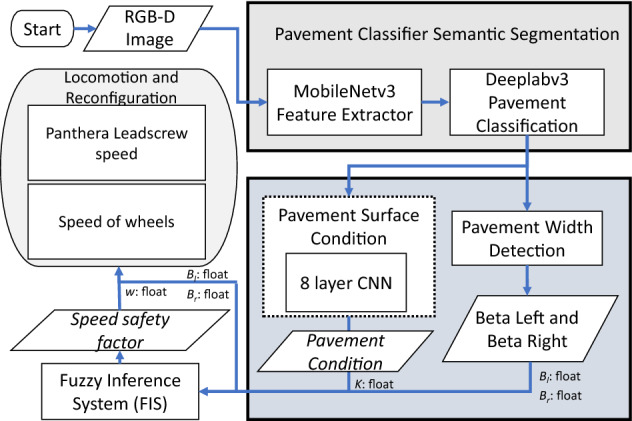
Vision pipeline.

The vision pipeline module executes three tasks: pavement type classification, pavement surface condition prediction, and pavement width estimation. The Fig. 3 shows the functional component of the vision pipeline. It comprises a DeepLabv3+ semantic segmentation framework, eight-layer CNN for pavement surface condition prediction, and pavement width estimation module. Here, the DeepLabv3+ is a critical component of the vision pipeline. The other two modules are built on top of the DeepLabv3+ framework. The vision pipeline ends with the output of the speed safety factor and the reconfiguration parameters^33^ for Panthera locomotion. The details of each component and its integration are described in the subsections.

### DeepLabv3+

**Figure 4:**
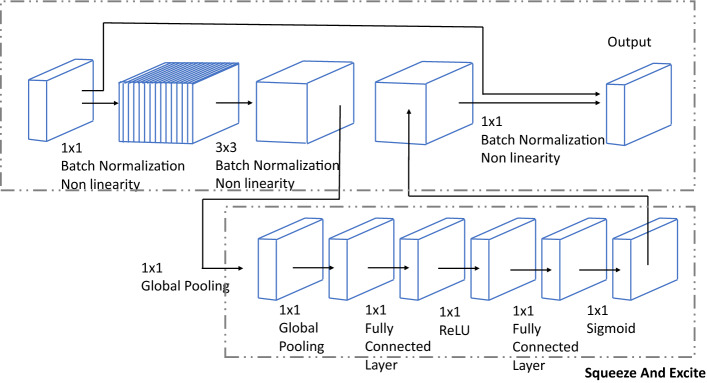
DeepLabv3+ semantic segmentation architecture.

Figure 4 shows the overview of the DeepLabv3+ semantic segmentation architecture. It comprised of the encoder–decoder function where the encoder function generates the feature map from the input images and decoder function gradually predict the object detail and spatial dimension of the objects.

#### Encoder

In DeepLabv3+, the encoder part consists of the backbone network, atrous separable convolution function, and atrous Spatial Pyramid Pooling (ASPP). In this work, MobileNetv3+ is configured as a backbone network. The layer detail of MobileNetV3+ is given in Table 2. In DeepLabv3+, the last convolution layer of MobileNetV3+ is replaced by an atrous separable convolution function to obtain the enlarged receptive field. Then, Atrous Spatial Pyramid Pooling (ASPP) function is applied on generated feature map, which applies four parallel convolution operations in feature map, including $1\times 1$ convolution and three $3\times 3$ convolutions dilation rates (6,12,18). In addition, ASPP applies Global Average Pooling (GAP) to the output features, a map from the last atrous block to obtained image-level features. In the end, the elements from all the branches are combined into a single vector via concatenation. This output is then convoluted with another $1\times 1$ kernel—using Batch Normalization (BN) and 256 filters.

**Table 2:** Specifications of MobileNetV3-Large.

C Input	Operator	exp size	#out	Squeeze-And-Excite	Non-Linearity	Stride
$224^{2} \times 3$	conv2d	–	16	No	h-swish	2
$112^{2} \times 16$	bneck, 3 $\times$ 3	16	16	No	ReLU	1
$112^{2} \times 16$	bneck, 3 $\times$ 3	64	24	No	ReLU	2
$56^{2} \times 24$	bneck, 3 $\times$ 3	72	24	No	ReLU	1
$56^{2} \times 24$	bneck, 5 $\times$ 5	72	24	Yes	ReLU	2
$28^{2} \times 40$	bneck, 5 $\times$ 5	120	40	Yes	ReLU	1
$28^{2} \times 40$	bneck, 5 $\times$ 5	120	40	Yes	ReLU	1
$28^{2} \times 40$	bneck, 3 $\times$ 3	240	80	No	h-swish	2
$14^{2} \times 80$	bneck, 3 $\times$ 3	200	80	No	h-swish	1
$14^{2} \times 80$	bneck, 3 $\times$ 3	184	80	No	h-swish	1
$14^{2} \times 80$	bneck, 3 $\times$ 3	184	80	No	h-swish	1
$14^{2} \times 80$	bneck, 3 $\times$ 3	480	112	Yes	h-swish	1
$14^{2} \times 112$	bneck, 3 $\times$ 3	672	112	Yes	h-swish	1
$14^{2} \times 112$	bneck, 5 $\times$ 5	672	160	Yes	h-swish	2
$7^{2} \times 160$	bneck, 5 $\times$ 5	960	160	Yes	h-swish	1
$7^{2} \times 160$	bneck, 5 $\times$ 5	960	160	Yes	h-swish	1
$7^{2} \times 160$	conv2d, 1 $\times$ 1	–	960	No	h-swish	1
$7^{2} \times 960$	pool, 7 $\times$ 7	–	–	No	–	1
$1^{2} \times 960$	conv2d, 1 $\times$ 1, No Batch Normalisation	–	1280	No	h-swish	1
$1^{2} \times 1280$	conv2d, 1 $\times$ 1, No Batch Normalisation	–	k	No	–	1

#### Decoder

The decoder module applies the upsampling function to retain the details (specifically object boundaries) from the low dimension feature map. In the decoder side, the multi-scale feature map (extracted from ASPP) is bilinearly upsampled by a factor of 4 and then concatenated with the corresponding low-level feature map obtained from MobileNetV3+ last convolution layer. Before concatenation, $1\times 1$ convolution is applied on a low-level feature map to reduce the number of channels. After the concatenation, a few $3 \times 3$ convolutions are applied to refine the features, followed by another simple bilinear upsampling of 4.

### Pavement surface condition prediction

The pavement surface condition is computed by CNN based classifier algorithm. It was cascaded with pavement segmentation framework through preprocessing function and took the segmented pavement region (200  $\times$  200  $\times$  3 ) as input. The classifier comprises of eight CNN layers, flatten layer, and two fully connected layers. At the end of each convolutional layer, the ReLU activation function and max pooling function are applied, where the max-pooling function reduces the dimensional of the feature map at each stage. The last three layers of the classifier frameworks are the flatten layers and two fully connected layers. The flatten layer converts the multi-dimension tensor data into a single dimension tensor and feeds into two fully connected (FC) layers. In FC layers, the ReLU activation function is applied on the first layer, and the SoftMax function is used on the second layer, which generates the probabilities output of pavement condition, *K*, which is used to drive the fuzzy inference system (FIS).

### Pavement width estimation

**Figure 5:**
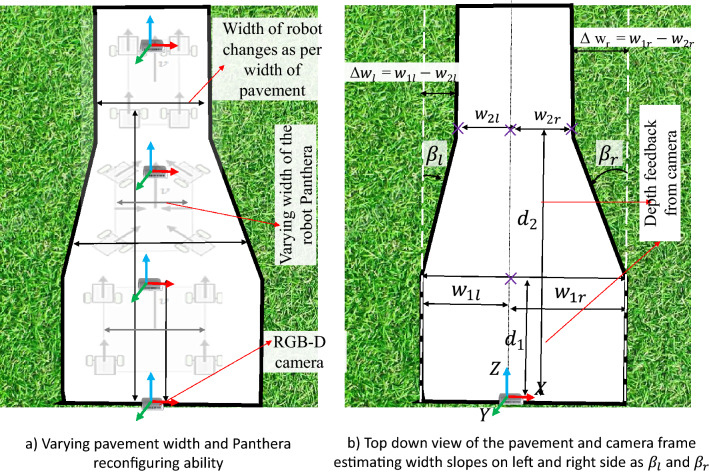
Pavement width estimation using vision feedback for reconfiguration parameters.

The pavement width was estimated from the segmented pavement region. It was computed for each frame captured from the Realsense RGB-D sensor. The segmented pavement region from DeepLabv3+ is used as an input to pavement width estimation function. The width estimation function uses the left and right fences of the segmented pavement region and corresponding point cloud data to measure the width of the pavement. The distance between the leftmost and rightmost point of the pavement 3D point cloud data x-axis, y-axis, and z-axis data were used to compute the pavement width. Finally, the euclidean 3D distance function was applied to each leftmost and rightmost point to compute the width of the pavement. After estimating the pavement width, it will derive vision feedback parameters for Panthera to perform reconfiguration as seen in Fig. 5a. The derivation of the vision feedback parameters $\beta _{l}$ and $\beta _{r}$ and the reduction of noise due to robot vibrations through filtering can be found in the works of^37^ and is visualized in Fig. 5b. The output *K*, and $\beta _{l}$, and $\beta _{r}$, which are the target heading angles of Panthera’s wheels, will be passed on to the FIS to control Panthera speed safety factor, *w*.

## Fuzzy inference system

**Figure 6:**
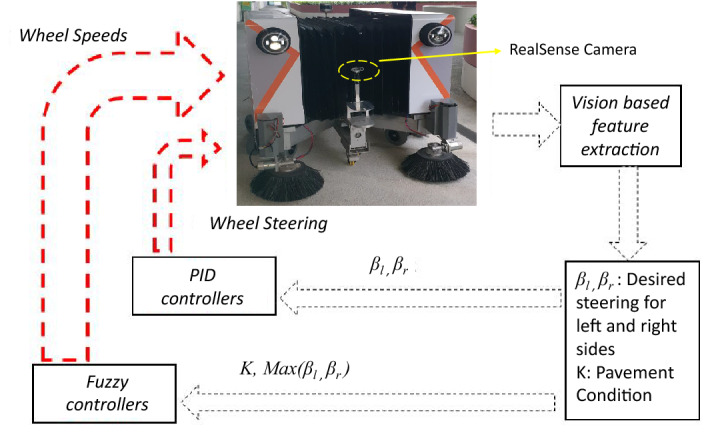
Controller architecture.

Figure 6 presents the overall control architecture. Here, the fuzzy inference system (FIS) is a part of the Panthera control system. It receives the input from the vision pipeline, including pavement condition, *K* and locomotion and reconfiguration parameters, $\beta _{l}$, and $\beta _{r}$^37^ to give the desired steering change for the wheels on the left and right side, respectively. Based on these factors, fuzzy logic is used to determine the safety factor, *w*, to apply to the robot speed without explicitly calculating the error variables. Given the desired headings, the conventional PID controllers control the steering. The robot’s speed safety factor, *w*, depends on all three parameters, and a fuzzy controller is proposed to modulate the speed. The fuzzy logic inference engine works with a simple rule base and input variables, which are fuzzy in nature. It aims to exploit the fuzzy sets and fuzzy inference method to incorporate safety into the Panthera robot.

**Figure 7:**
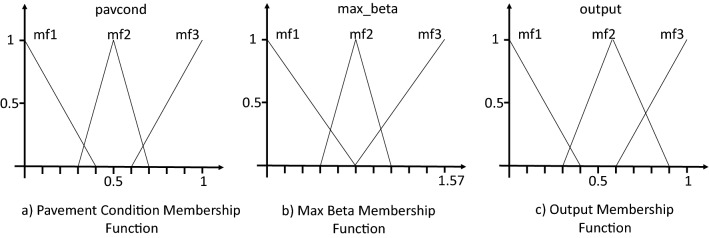
Membership functions.

Fuzzy Controller: The controller exploits a Mamdani fuzzy inference engine. The pavement condition data received from the vision module is modeled as three Gaussian membership functions; bad (mf1), moderate (mf2), and good (mf3). The steering requirement is calculated from the pavement width data. The bigger steering requirement between $\beta _{l}$ and $\beta _{r}$ is taken as another input and modeled in the same manner as small, moderate, and large angles. The speed safety factor, *w*, is captured using a similar approach. The details of the membership functions and the set of nine rules are presented in Fig. 7 and Table 3 respectively.

**Table 3:** Fuzzy rules.

Rule	pavcond	max_beta	speed safety factor
1	Bad	High	Slow
2	Bad	Medium	Slow
3	Bad	Small	Slow
4	Moderate	High	Slow
5	Moderate	Medium	Normal
6	Moderate	Small	Normal
7	Good	Small	High
8	Good	High	Normal
9	Good	Medium	High

## Experimental results

This section describes the experimental setup procedure and outcome of the proposed vision pipeline system. The experimental setup procedure includes collecting the dataset image and labeling, training the model with labeled images, and evaluating the trained model using test images and real-time video stream.

### Dataset preparation

Panthera is developed for pavement sweeping tasks in public pavement spaces. Hence, the dataset images are collected from public pavement spaces in Singapore, including national parks, park connectors, residential parks, and school parks. The collected datasets are categorized into three classes such as concrete pavement, paver block pavement (a stone, brick, or block used for paving a surface), and roads to train the segmentation model. Furthermore, to train the pavement surface condition algorithm, the dataset were labelled into three category which include bad, moderate, and good as shown in Figs. 26, 27 and 28 respectively. Intel RealSense D435 stereo vision sensor was used to collect dataset images. It was mounted on a bicycle to collect the images from different public pavement spaces at 95 cm height to get the same field of view as Panthera. After collecting the initial set of images of the three pavement types, the data augmentation process is applied to the ordered dataset, which involves adjusting the image orientation, varying brightness, adjusting the scale of the images, etc. This process will help control the over-fitting issue and make the model more robust. Then, the labeled image data were randomly divided into the train and test dataset according to the hierarchical sampling method.

### Training

Transfer learning techniques were adopted to train the DeepLabv3+ pavement segmentation model, where cityscapes pre-trained weights files were used to fine-tune the model. The framework was trained with 2000 images in each class and used the following training parameter learning rate: 0.001, weight decay: 0.0005, momentum: 0.9, and batch size: 10. The eight-layer CNN framework was trained from scratch with Adam optimizer and used the segmented pavement region as a training dataset. The weight was initialized with the uniform method and applied the learning rate: 0.001, weight decay: 0.0005, momentum: 0.9, and batch size: 64. Both models were trained on NVIDIA RTX 3080 graphic processing unit enabled workstation and trained on GPU mode.

### Offline and real-time evaluation test

**Figure 8:**
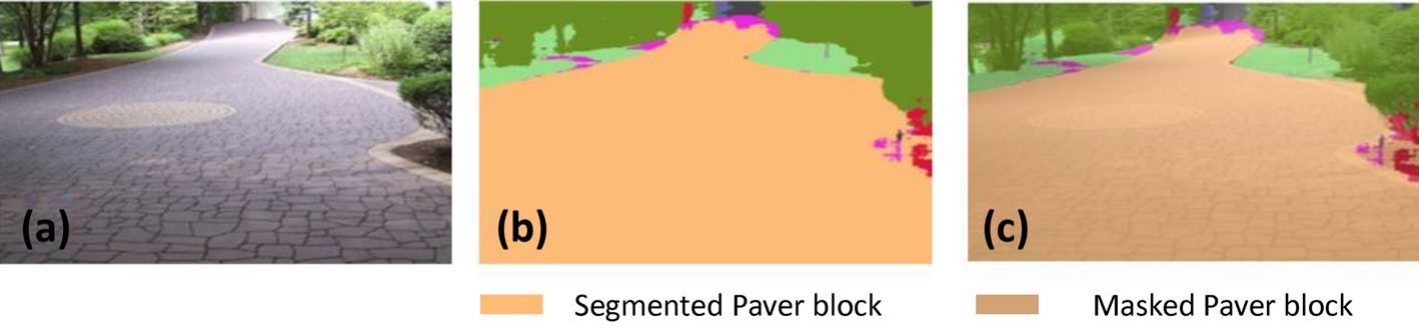
Offline results: paver block.

**Figure 9:**
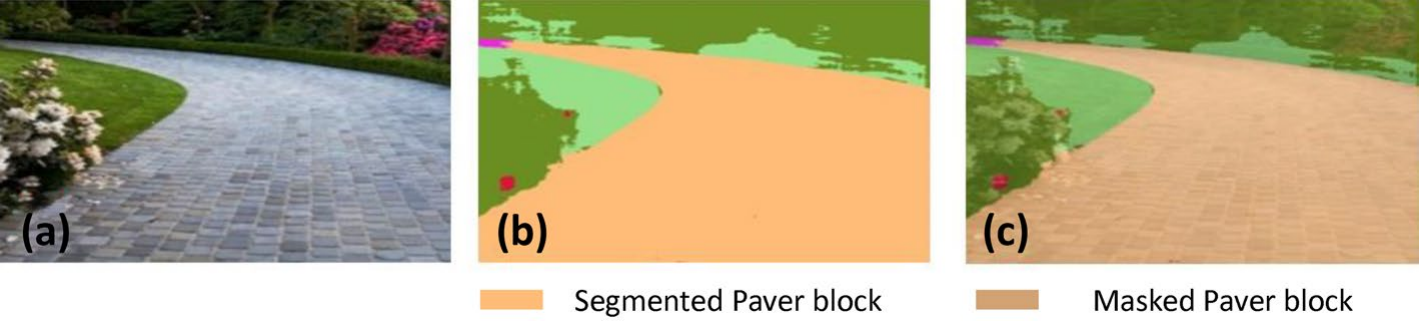
Offline results: paver block.

**Figure 10:**
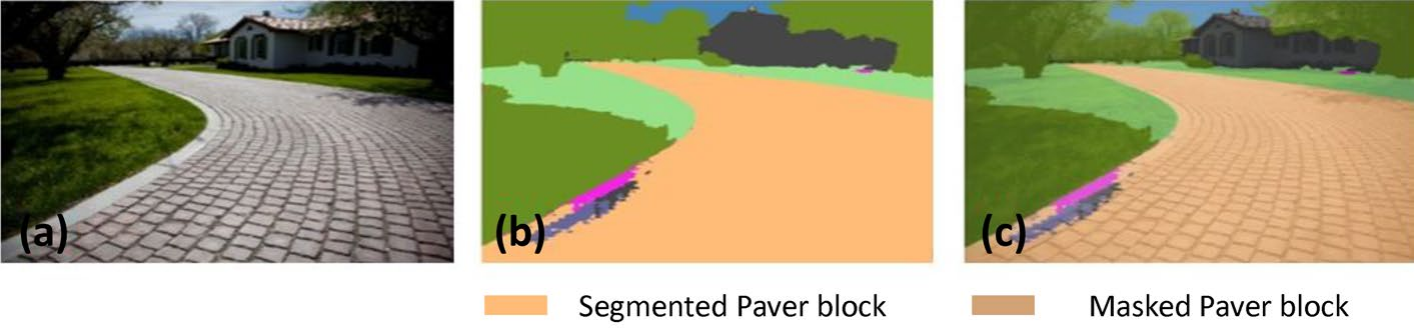
Offline results: paver block.

**Figure 11:**
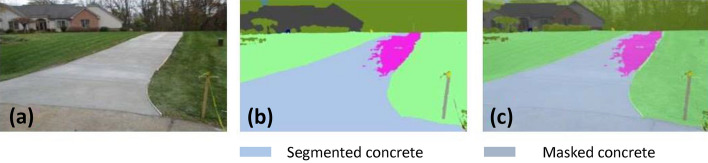
Offline results: concrete.

**Figure 12:**
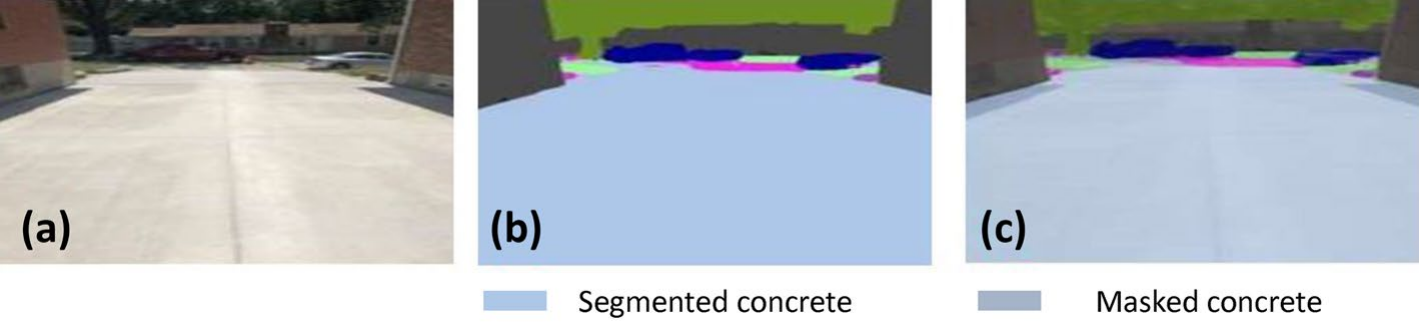
Offline results: concrete.

**Figure 13:**
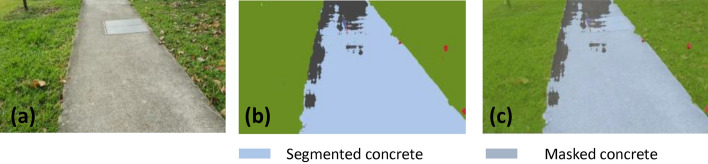
Offline results: concrete.

**Figure 14:**
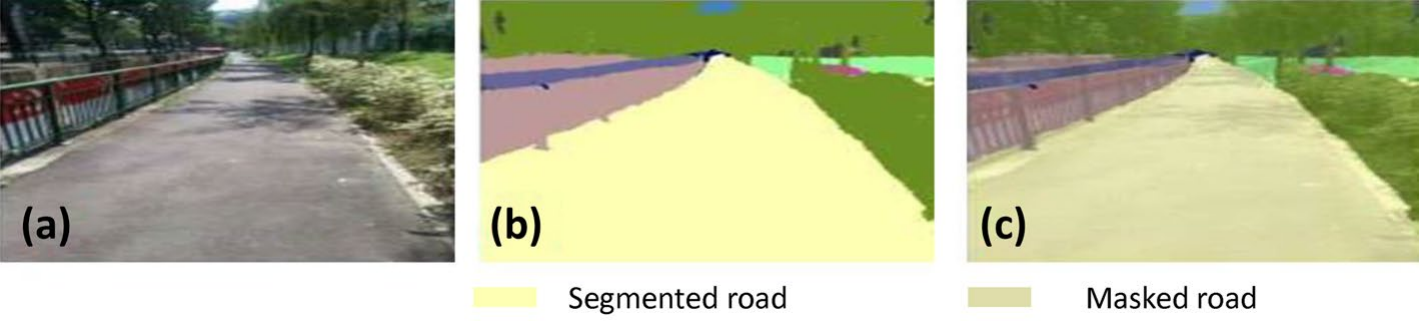
Offline results: road.

**Figure 15:**
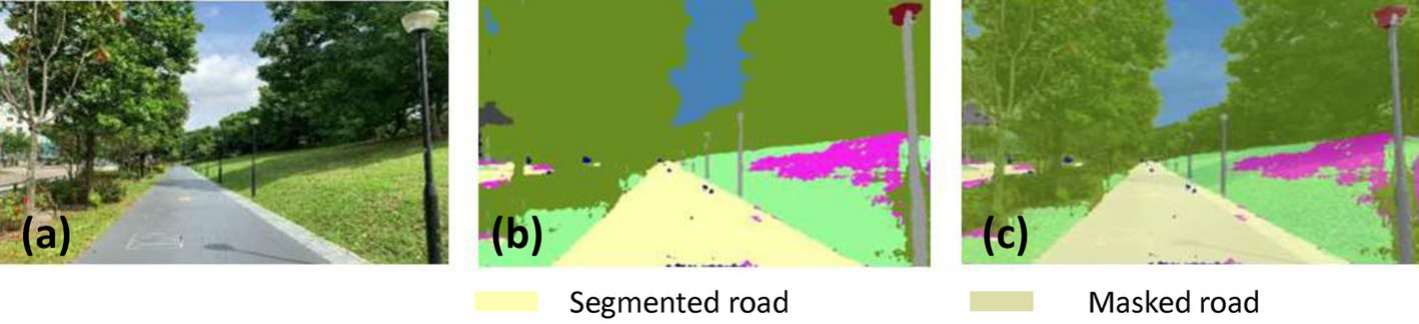
Offline results: road.

**Figure 16:**
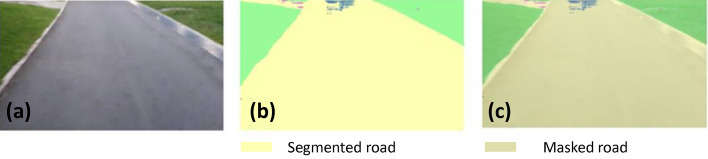
Offline results: road.

**Figure 17:**
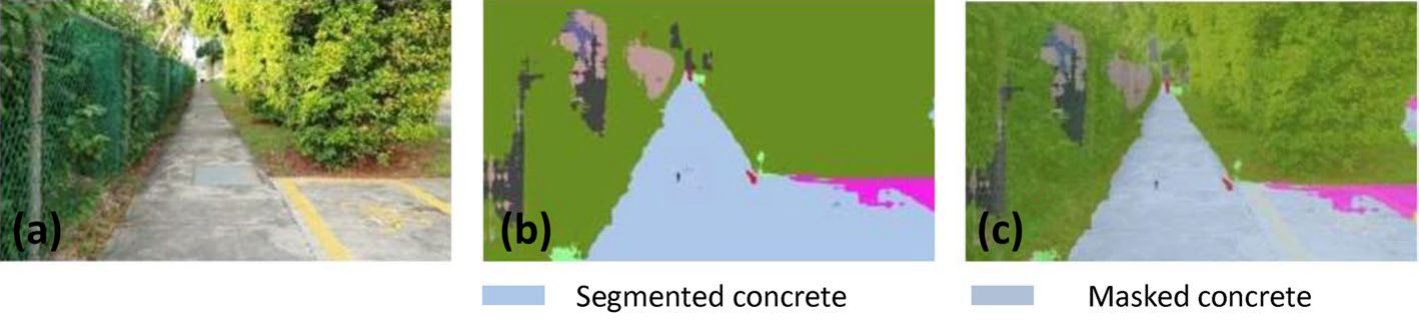
Online results: concrete.

**Figure 18:**
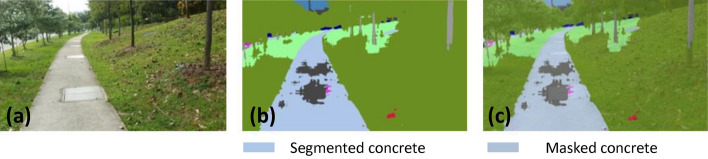
Online results: concrete.

**Figure 19:**
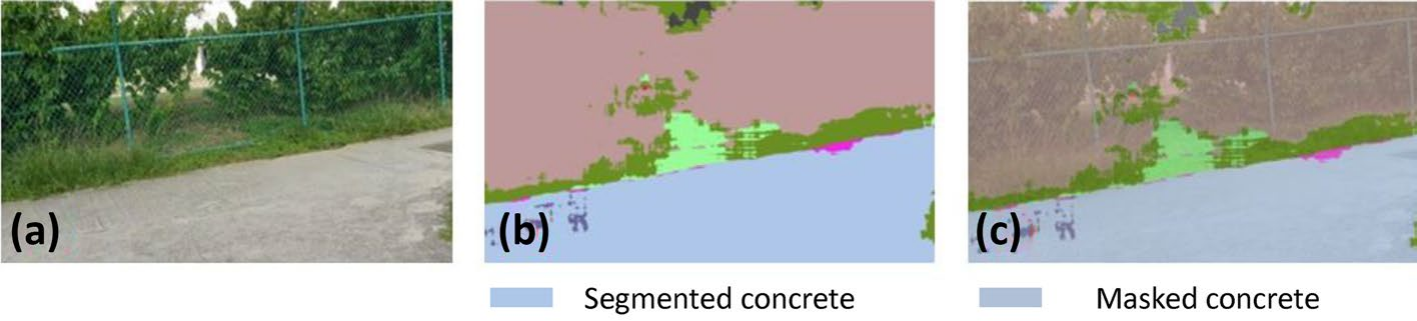
Online results: concrete.

**Figure 20:**
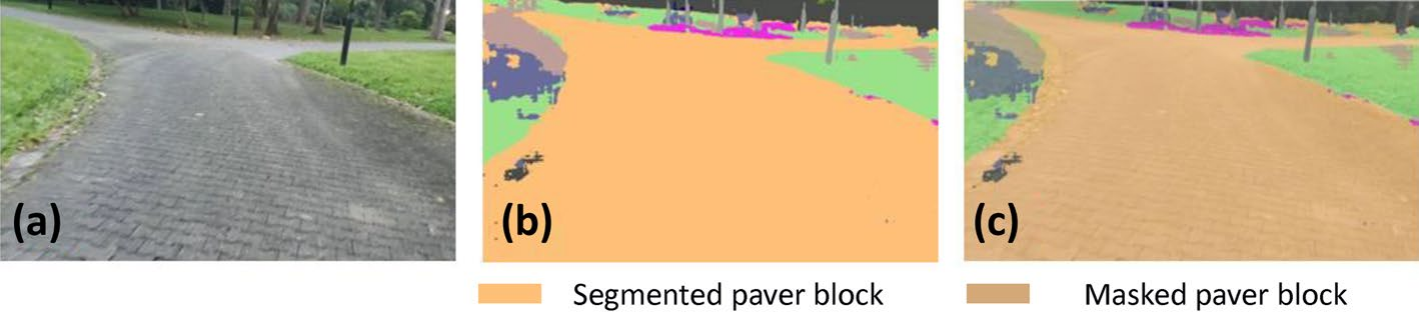
Online results: paver block.

**Figure 21:**
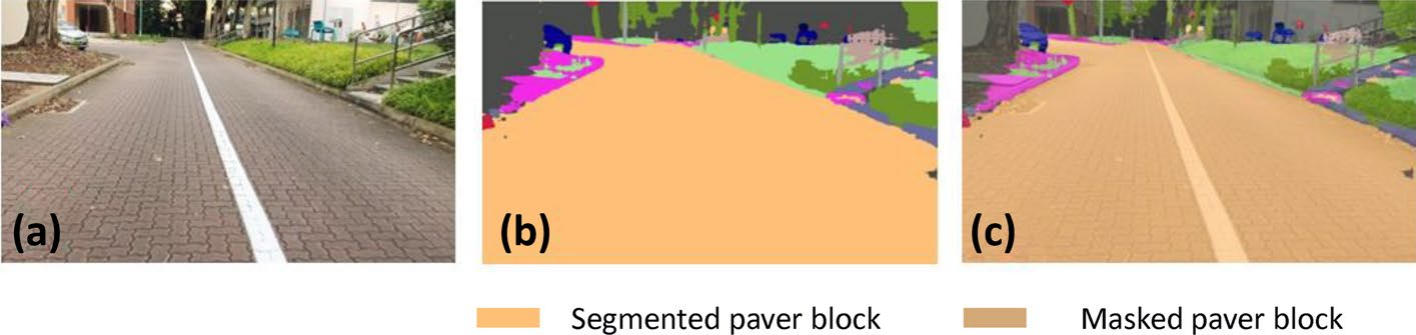
Online results: paver block.

**Figure 22:**
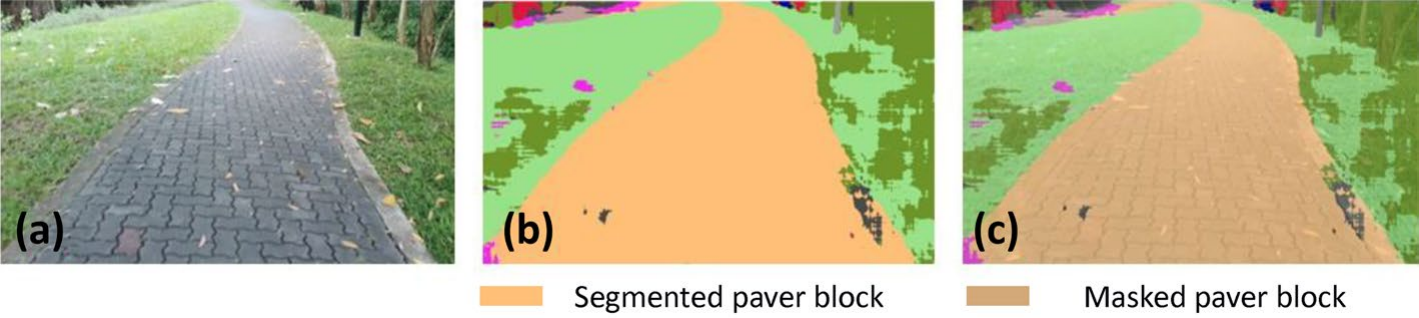
Online results: paver block.

**Figure 23:**
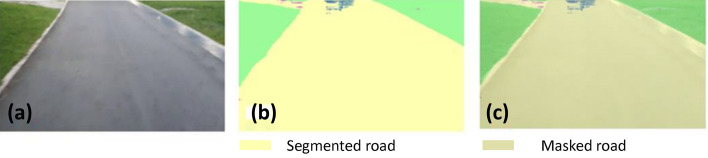
Online results: road.

**Figure 24:**
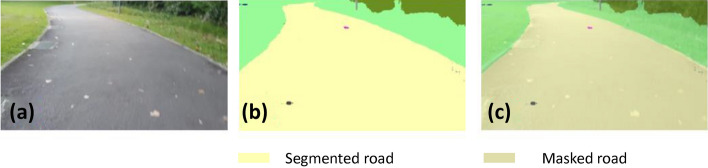
Online results: road.

**Figure 25:**
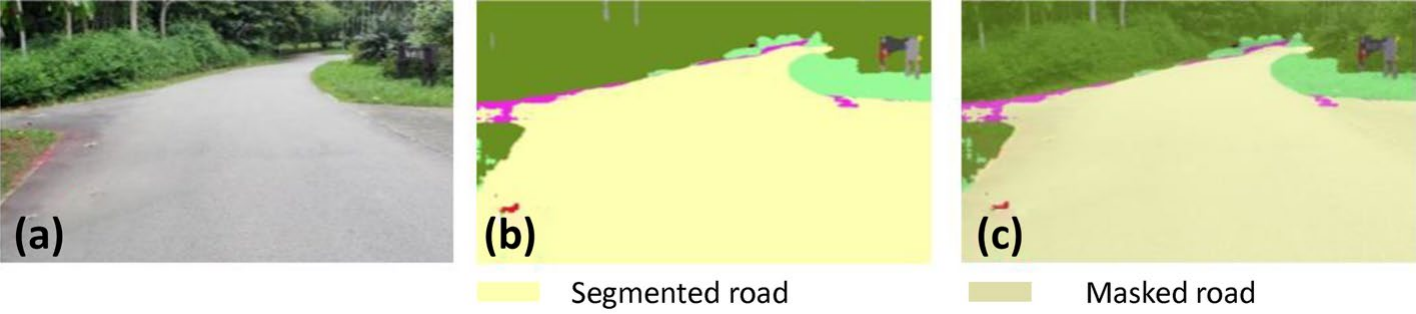
Online results: road.

After training, the segmentation and detection framework performance was assessed through test images and real-time video streams. Totally 200 images were used for each class in test image dataset and its segmentation and classification accuracy was estimated through statistical measure parameter. To carry out the evaluation test, the trained model was loaded into NVIDIA RTX 3080 graphic processing unit enabled workstation and tested with collected pavement images. Figures 8, 9, 10, 11, 12, 13, 14, 15 and 16 shows the segmentation and classification results of test image dataset. Figures 17, 18, 19, 20, 21, 22, 23, 24 and 25 shows the online experimental results of pavement segmentation model. In all the Figures, (a) represents the Red Green Blue (RGB) image, (b) represents the segmented image and (c) represents the segmented masked image. The color schemed for paver block, concrete and road in (b) and (c) is consistent throughout the paper. Table 4 indicates the performance analysis report for offline and online test. In real time field trial , concrete, paver block and road pavement video streams are taken as the online input data to the model. In each class, a 100 m pavement was captured from Panthera robot perspective using RealSense D435 Camera. The image resolution of the RealSense D435 used is $640\times 480$ where the processing node runs at about 20 frames per second.

**Table 4:** Performance analysis for pavement segmentation.

Test model	Pixel accuracy (Average)	mean-IOU	Dice score
Offline test	89.25	88.22	88.27
Online test	86.79	85.54	86.12

The experiment results indicate that the segmentation algorithm accurately segmented the pavement and its boundary region and scored an average of 89.25% pixel classification accuracy for the test image dataset and 86.79% pixel classification accuracy for real-time collected pavement streaming video input which took approximately 10 ms to segment and classify each image. This analysis shows that the pixel classification accuracy for a real-time field trial is lower than the test image dataset. It is due to various environmental factors, such as jerks in locomotion, shadows, lighting conditions, etc.

### Pavement surface condition and width estimation

**Figure 26:**
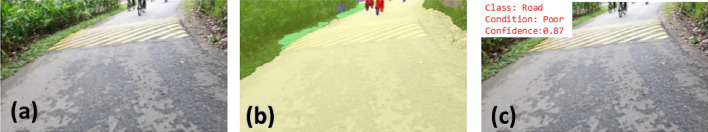
Pavement condition: bad.

**Figure 27:**
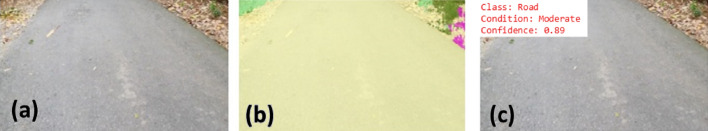
Pavement condition: moderate.

**Figure 28:**
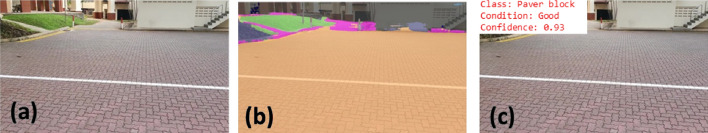
Pavement condition: good.

The Fig. 26, 27 and 28 shows the pavement surface condition prediction result computed from segmented pavement region and Table 5 shows the statistical measures results for pavement surface condition model computed through confusion matrix parameters. In this experimental analysis, it observed that the surface condition classification model obtained average classification accuracy of 92.93%, and its prediction confidence score range is 87–94% respectively. Moderate class precision and accuracy is slightly lower than Good and Poor class as it has two boundaries for the classification whereas Good and Poor class only have one boundary.

**Table 5:** Statistical measures analysis for pavement’s surface condition.

Class	Precision	Recall	F1	Accuracy
Good	93.44	92.89	92.65	93.17
Moderate	92.12	90.31	93.23	91.89
Poor	94.12	93.32	92.97	93.74

**Figure 29:**
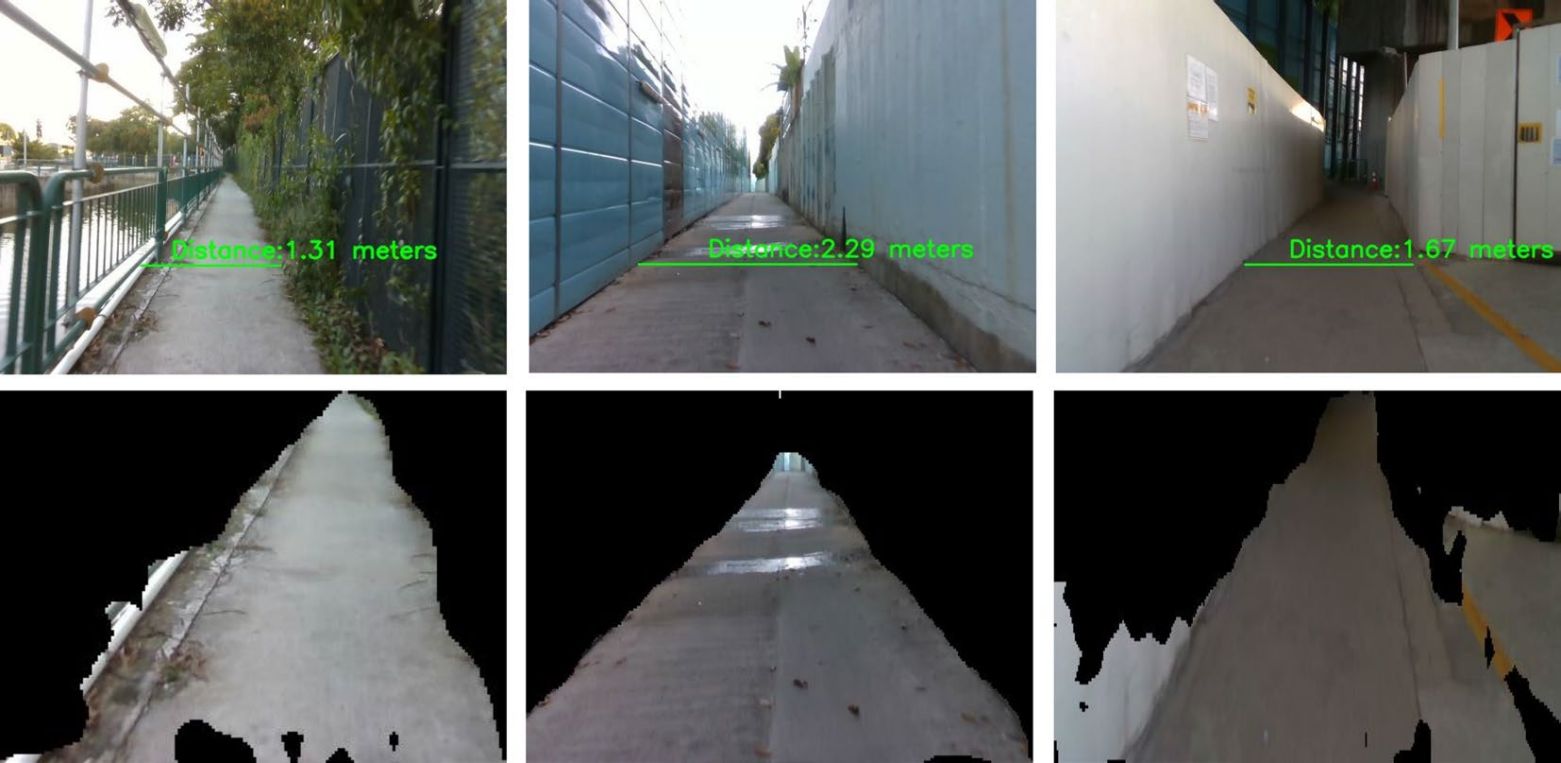
Offline results: pavement width estimation from pavement segmented region.

**Figure 30:**
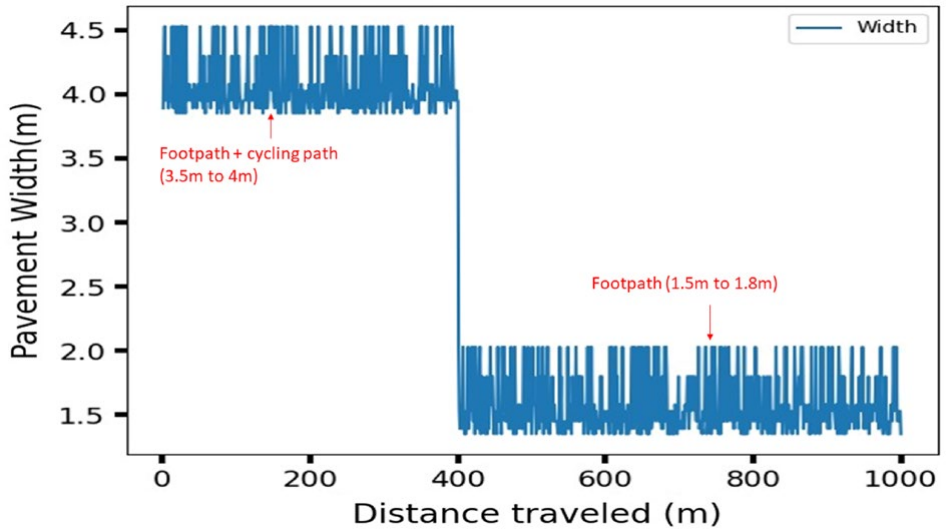
Pavement width estimation.

Figure 29 shows the pavement width estimation results computed from segmented pavement region part and Fig. 30 shows the pavement width graph for two different pavements computed for 1 km. To predict a one-meter pavement’s width, 100 frames results were considered, computed based on robot operation speed of 700 m per hour. At the end of the vision pipeline, the generated surface condition and pavement width information are passed to the FIS to output the speed safety factor of the robot.

### Fuzzy controller output

**Figure 31:**
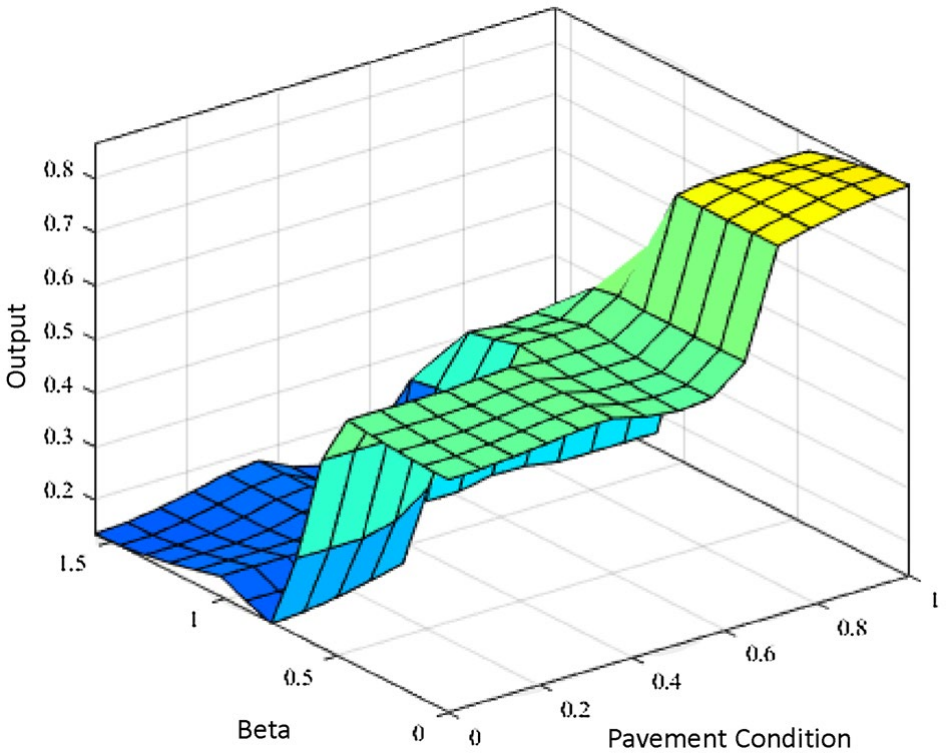
Surface plot for output speed safety factor.

Based on the membership function and rules, the output of the fuzzy controller is shown in a surface plot given in Fig. 31. Speed of Panthera is multiplied by the output of the fuzzy controller speed safety factor, *w*, to consider the vision pipeline parameters *K*, $\beta _{l}$, $\beta _{r}$ where *K* is assigned based on the pavement classification. It can be observed that the robot’s speed is maximum only when pavement condition is good and steering requirement is less. If the steering requirement is high, the speed is always minimum irrespective of pavement condition. This feature also suits the dynamic reconfiguration while avoiding various obstacles during motion. Similarly, the speed is minimum when the pavement condition is bad, irrespective of steering requirement. Panthera speed is adjusted for safety during bad pavement conditions and large steering requirements through the fuzzy controller.

### Comparison analysis

**Figure 32:**
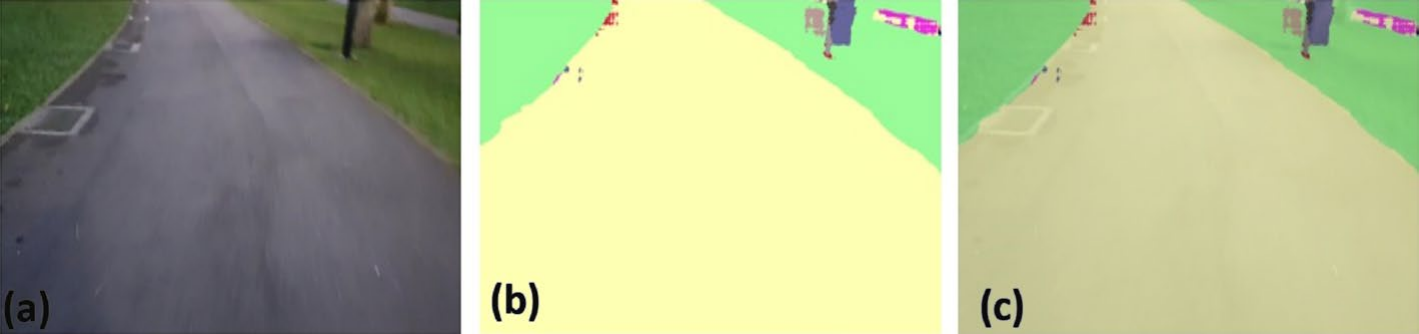
DeepLabv3+ segmentation results.

**Figure 33:**
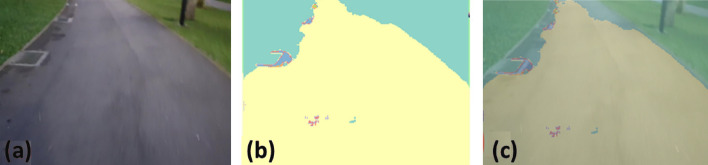
Unet segmentation results.

**Figure 34:**
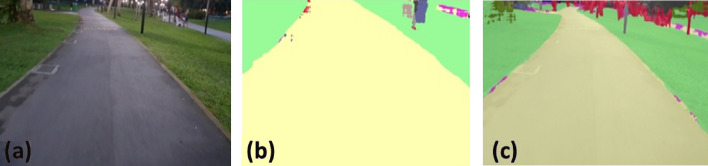
DeepLabv3+ segmentation results: pavement with night mode.

**Figure 35:**
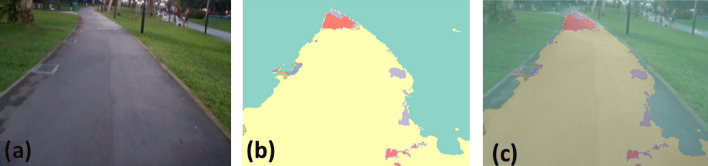
Unet segmentation results: pavement with night mode.

**Figure 36:**
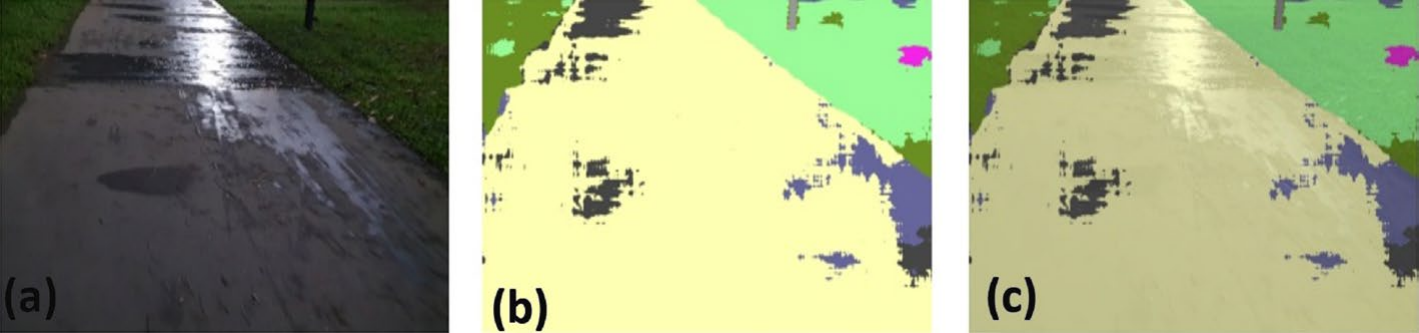
DeepLabv3+ segmentation results: pavement with water puddle.

**Figure 37:**
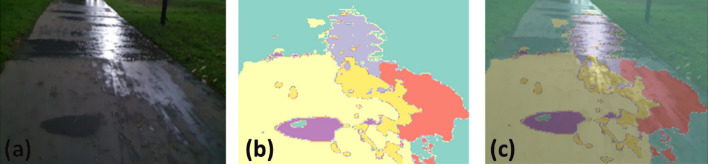
Unet segmentation results: pavement with water puddle.

The DeepLabv3+ semantic Segmentation model was compared with the UNET Segmentation model. Here, the UNET model was trained with the same dataset till convergence and uses pixel classification accuracy and inference time as evaluation metrics. Figures 32, 33, 34, 35, 36 and 37 shows the segmentation result of DeepLabv3+ and UNET framework.

The comparison analysis indicates that DeepLabv3+ outperforms UNET in terms of segmentation accuracy and inference time. In UNET, the segmentation performance was relatively poor for night mode collected images and water puddles in the driveway. In this comparison analysis, DeepLabv3+ scored 92% classification accuracy and took 10 ms inference time. On the other hand, UNET scored 87% classification and took 22 ms for inference one image.

A short summary of the advantages and limitations for the proposed algorithm are as follows:

- The advantages:
  - Safer autonomous pavement sweeping self-reconfigurable robots which changes speed based on a speed safety factor, *w*, that adapts to varying reconfiguration parameters and pavement conditions.
  - High level of accuracy of pavement segmentation allows self-reconfigurable robot to more accurately identify the reconfiguration parameters $\beta _{l}$ and $\beta _{r}$.
  - Inference time of the vision pipeline is low.
- The limitations:
  - Models need to be retrained for different pavement types such as wood.
  - Accuracy might be affected by environmental changes such as heavy rain due to lack of visibility
  - Proposed algorithm focuses heavily on pavement segmentation and condition classification. Addition of other classes such as people, animals and vehicles might reduce the model accuracy.

## Conclusions

This work proposed the deep learning-based vision pipeline for the reconfigurable pavement sweeping robot Panthera. Through a vision pipeline, the robot has been able to identify the pavement type, pavement condition, reconfiguration parameters to allow the robot to adapt to pavements of changing width with a safety factor based on fuzzy control. The efficiency of the deep learning-based vision pipeline was evaluated with a real pavement testbed, and its detection accuracy was estimated with standard performance metrics. The experimental results indicate that the vision pipeline classifies pavement segmentation and surface condition with 88% and 93% pixel-level classification accuracy. Furthermore, the proposed system was tested in varying lighting conditions and pavement types in Singapore and ensured that the model segmentation and detection accuracy were more stable with various pavement conditions. From the high level of accuracy in the vision pipeline, reconfiguration parameters and safety factors can be derived accurately for the safer operation of the Self-reconfigurable robot Panthera.

## Data Availability

The datasets used and/or analysed during the current study available from the corresponding author on reasonable request.
